# Efferocytosis: Burying cell corpses to regulate tolerance and immunity

**DOI:** 10.18632/oncotarget.4402

**Published:** 2015-06-09

**Authors:** Maziar Divangahi

**Affiliations:** Departments of Medicine, Microbiology & Immunology and Pathology, McGill International TB Centre, McGill University Health Centre, Centre for Translational Biology, Meakins-Christie Laboratories, Montreal, Canada

It is remarkable that every second approximately one million of our cells die via apoptosis, yet the collateral damage incurred (e.g. onset of autoimmune disease) remains relatively rare [1]. Disposal of this massive number of cell corpses evolved to not only maintain homeostasis but also prevent dissemination of intracellular pathogens and promote host defense. For instance, during infection with *Mycobacterium tuberculosis* (*Mtb*), processing of infected apoptotic cells by macrophages [2] and dendritic cells (DC) [3] enhances anti-bacterial innate and adaptive immune responses. Uptake of apoptotic bodies by phagocytes to maintain or resume homeostatic conditions and prevent pathology is mediated through an elegant process called efferocytosis (Efferre in Latin means “to bury”).

Recent studies have shown that efferocytosis differs from classical phagocytosis in several respects including the use of distinct receptors, bridging proteins and signaling pathways. Specifically, efferocytosis depends on three main steps: (1) a “find-me” signal or the release of soluble mediators from apoptotic cells that recruit phagocytes; (2) an “eat-me” signal including exposure of a unique set of engulfment ligands on the surface of apoptotic cells leading to their recognition by phagocytes; and (3) the formation of the efferosome (a specific phagosome) and its degradation [4]. The best characterized eat-me signal is the exposure of phosphatidylserine (PS) on the surface of apoptotic cells. However, PS alone is not sufficient in triggering engulfment and other eat-me signals are required for optimum efferocytosis.

Annexin 1 is a member of the annexin family first shown to co-localize with PS on the surface of apoptotic cells promoting efficient efferocytosis under physiological conditions in *Caenorhabditis elegans* [5]. Consistently, we recently demonstrated that this pathway is also operational in mammalian cells and plays an essential role in immunity to *Mtb*. During *Mtb* infection, annexin 1 in DCs increased efferocytosis and enhanced the capacity of antigen-presenting machinery leading to more efficient cross-presentation of antigens to CD8 T cells. Considering that annexin 1 is an essential eat-me signal under both physiological and immunogenic conditions, how does the immune system distinguish between these settings to promote host defense and prevent autoimmunity? We envision that apoptotic cells expose two sets of “eat-me” signals on their plasma membrane. One set is common to both immunogenic and tolerogenic apoptotic cells facilitating recognition/internalization under both conditions (e.g. PS or annexin 1). The second set is more unique to immunogenic apoptotic cells that are undergoing death due to infection and potentially exposing differential levels of surface markers involved in anti-microbial immunity. For instance activation of pattern recognition receptors have been shown to be critical for induction of immunogenic apoptosis during mycobacterial infection [6]. Certainly, further experiments are needed to identify the molecular mechanisms involved in this process.

In addition, the machinery of the immunogenic efferosome in macrophages versus DC may differ. For instance, *Mtb*-infected macrophages that die via apoptosis are rapidly efferocytosed by uninfected macrophages, which ultimately results in lysosome fusion and destruction of *Mtb* [2]. However, the mechanisms by which antigens derived from the efferosome are acquired by DC are still poorly understood. Our data suggest that annexin 1 is also involved in autophagy which may facilitate access to antigens for cross-presentation [3]. Future studies are necessary to assess the fate of efferosome in DC.

As we have evolved with such a refined immune system, pathogens have also developed sophisticated mechanisms in-step to deal with our immunity including hijacking process involved in cell death. Although efferocytosis of apoptotic *Mtb*-infected macrophages by macrophages/DC is beneficial to the host, some other intracellular pathogens have evolved to subvert efferocytosis for their own survival. For instance, efferocytois of apoptotic *Leishmania major*-infected neutrophils by macrophages may promote infection as the bacteria appears to utilize efferocytosis as “Trojan Horse” for getting access into the macrophages for replication [7]. Thus the arms race between pathogens and hosts will persist indefinitely.
